# Increasing shape modelling accuracy by adjusting for subject positioning: An application to the analysis of radiographic proximal femur symmetry using data from the Osteoarthritis Initiative^☆^

**DOI:** 10.1016/j.bone.2014.01.003

**Published:** 2014-04

**Authors:** C. Lindner, G.A. Wallis, T.F. Cootes

**Affiliations:** a Centre for Imaging Sciences, The University of Manchester, Manchester M13 9PT, UK; b Wellcome Trust Centre for Cell Matrix Research, The University of Manchester, Manchester M13 9PT, UK

**Keywords:** Statistical shape model, Hip morphology, Preoperative planning, Total hip arthroplasty, Osteoarthritis, Osteoporosis

## Abstract

In total hip arthroplasty, the shape of the contra-lateral femur frequently serves as a template for preoperative planning. Previous research on contra-lateral femoral symmetry has been based on conventional hip geometric measurements (which reduce shape to a series of linear measurements) and did not take the effect of subject positioning on radiographic femur shape into account. The aim of this study was to analyse proximal femur symmetry based on statistical shape models (SSMs) which quantify *global* femoral shape while also adjusting for differences in subject positioning during image acquisition. We applied our recently developed fully automatic shape model matching (*FASMM*) system to automatically segment the proximal femur from AP pelvic radiographs to generate SSMs of the proximal femurs of 1258 Caucasian females (mean age: 61.3 SD = 9.0). We used a combined SSM (capturing the left and right femurs) to identify and adjust for shape variation attributable to subject positioning as well as a single SSM (including all femurs as left femurs) to analyse proximal femur symmetry. We also calculated conventional hip geometric measurements (head diameter, neck width, shaft width and neck-shaft angle) using the output of the *FASMM* system. The combined SSM revealed two modes that were clearly attributable to subject positioning. The average difference (mean point-to-curve distance) between left and right femur shape was 1.0 mm before and 0.8 mm after adjusting for these two modes. The automatic calculation of conventional hip geometric measurements after adjustment gave an average absolute percent asymmetry of within 3.1% and an average absolute difference of within 1.1 mm or 2.9° for all measurements. We conclude that *(i)* for Caucasian females the *global* shape of the right and left proximal femurs is symmetric without isolated locations of asymmetry; *(ii)* a combined left–right SSM can be used to adjust for radiographic shape variation due to subject positioning; and *(iii)* adjusting for subject positioning increases the accuracy of predicting the shape of the contra-lateral hip.

## Highlights

• Radiographic proximal femur symmetry is analysed using statistical shape models (SSMs).
• An SSM-based method to adjust for subject positioning effects on radiographic proximal femur shape is proposed.
• The *global* shape of the right and left proximal femurs is symmetric without isolated locations of asymmetry.
• A combined left–right proximal femur SSM can be used to adjust for radiographic shape variation due to subject positioning.
• Adjusting for subject positioning increases the accuracy of predicting the shape of the contra-lateral hip.

## Introduction

Anteroposterior (AP) pelvic radiographs are widely used in clinical practice for the assessment of skeletal disorders such as hip osteoarthritis or osteoporosis. In total hip arthroplasty, preoperative templating based on AP pelvic radiographs is an important step in predicting features such as implant size, position and orientation [1–3]. Depending on the severity of disease, for example in cases of arthritic hips or osteoporotic fractures, the AP view of the affected side may not provide sufficient information to serve as a template. In such cases, the shape of the contra-lateral femur frequently serves as a template for orthopaedic surgery planning.

Several studies have been undertaken to analyse bilateral femoral symmetry in humans that have suggested that such symmetry can be assumed [4–8]. However, past studies have either focussed on non-geometric aspects of femoral symmetry (e.g. structural properties), or have used pre-defined conventional hip geometric measurements (e.g. femoral head diameter or neck-shaft angle) and sample sizes have been small. Conventional hip geometric measurements reduce shape to a series of linear measurements rather than taking global shape into account. More recently, statistical shape models (SSMs) have been introduced for detailed morphometric analysis of *global* bone shape [9–13]. SSMs describe every shape by the sum of a mean shape and a linear combination of a number of shape modes which allows the quantification of overall proximal femur shape for each subject. In analyses of hip morphometry, SSMs have been successfully used to identify key features of bone shape that contribute to the progression of radiological hip osteoarthritis, to predict osteoporotic hip fractures, and to analyse genetic contributors to hip osteoarthritis [10,11,14–16].

We have recently developed a fully automatic shape model matching (*FASMM*) system to rapidly and accurately segment the proximal femur from AP pelvic radiographs and to represent the shape of the proximal femur using an SSM [17]. The generated SSM allows the quantitative description of global proximal femur morphology. However, as AP pelvic radiographs only give a two-dimensional projection of what is a three-dimensional structure, the projected radiographic shape will differ depending on the positioning of the subject (e.g. pelvic or internal/external leg rotation during image acquisition). Hence, relying on measurements taken directly from the AP projected view without any adjustment for subject positioning may lead to an incorrect representation of the actual shape.

The specific aims of this study were therefore to: (1) analyse the symmetry of the left and right proximal femurs via using SSMs to quantify *global* proximal femur morphology; (2) develop an SSM-based method that adjusts for subject positioning during image acquisition; and (3) assess the impact of subject positioning on the projected radiographic shape via the analysis of hip geometric measurements automatically calculated from the output of the *FASMM* system.

## Materials and methods

### Dataset

Data used in the preparation of this article were obtained from the Osteoarthritis Initiative (OAI) database, which is available for public access at http://www.oai.ucsf.edu/. We used radiographs from release 0.E.1 of the ‘Images’ dataset. Clinical data came from version 0.2.2 of each of the clinical datasets ‘Enrollees’, ‘Subject Characteristics’, ‘Medical History’, and ‘Physical Exam’. Demographic data for each subject included body mass index (BMI) and age at enrolment.

Baseline AP pelvic radiographs from 4796 subjects (2804 females and 1992 males) were available. For our analyses, we selected the larger subgroup of 2124 Caucasian females to limit the variation that might occur due to the differences in hip joint shape between males and females [18,19,6,20] or between ethnic groups [21,22] and thereby increase the likelihood of identifying shape variation that was attributable to subject positioning. From this dataset of 2124 Caucasian females, we also excluded subjects who had had hip replacement surgery at baseline or with a self-reported diagnosis of hip osteoarthritis at baseline or at 48 months follow up as recorded in the OAI database. The reason for exclusion based on these latter criteria was that the *FASMM* system had been trained previously on OA-unaffected proximal femurs [17,20]. Radiographic scoring of all radiographs is not currently available for the OAI radiographs and so exclusion on the basis of radiographic osteoarthritis was not undertaken. Hence, it is possible that subjects with radiographic evidence of osteoarthritis in the absence of a diagnosis of osteoarthritis may have been included in our dataset. There was no evidence, however, that this had a negative impact on the performance of the *FASMM* system (see Section 3.1). Application of these exclusion criteria reduced the dataset to 1610 baseline AP pelvic radiographs of which 1282 included both the left and right proximal femurs without any occlusions and were selected for further study. Table 1 summarises key features of relevant medical history for the 1282 subjects included in this study.

### The *FASMM* system

**Fig. 1:**
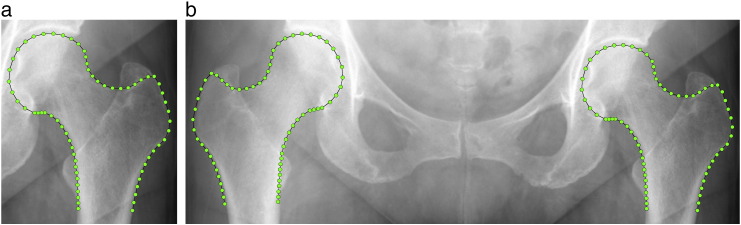
Segmentation examples showing (a) the single proximal femur model using 65 points and (b) the combined model using 130 points.

We used the previously described *FASMM* system to accurately and fully automatically segment the proximal femur in pelvic radiographs [17]. As previously described [20], the system was trained on 1105 AP pelvic radiographs from subjects recruited in Stage 2 of The arcOGEN Consortium study [23]. The *FASMM* system segments the proximal femur by first detecting it in the radiograph and then outlining its contour using 65 points (see Fig. 1a) that are placed in consistent positions across all images. The system uses a front-view femur model that excludes both the lesser and greater trochanters.

As previously described [20], the contour points returned by the *FASMM* system are used to represent the shape of the proximal femur as an SSM. This provides a *global* representation of shape rather than reducing shape to a series of linear measurements which enables the analysis of shape variation across datasets. Based on a number of points in a set of images, an SSM is trained by applying principal component analysis to the aligned shapes [24]. This yields a linear model of shape variation which represented the position of each point *l* using

(1) $$x_{l}=T_{\theta }\;\left({\bar{x}}_{l}+P_{l}b+r_{l}\right)$$

where ${\bar{x}}_{l}$ was the mean position of the point in a suitable reference frame, ***P***_***l***_ was a set of modes of variation, ***b*** were the shape model parameters, ***r***_*l*_ allowed small deviations from the model, and *T*_*θ*_ applied a global transformation (e.g. similarity) with parameters *θ*. All modes of variation in ***P***_***l***_ are orthogonal and every mode defines a pattern of shape variation. The first mode in ***P***_***l***_ accounts for the largest amount of shape variation across the dataset, the second mode for the largest amount of shape variation still remaining and so on. For the purpose of analysis of shape variation, the inclusion of the modes that describe 95% of the overall shape variation is accepted in the field as an appropriate cut-off to minimise noise [25–27].

### Adjusting for subject positioning

We applied the *FASMM* system to fully automatically segment the left and right proximal femurs from the radiographs of all subjects. We used the output of the *FASMM* system (i.e. 65 contour points per proximal femur per subject) to generate a combined SSM that included both the left and right proximal femurs at the same time and hence was derived from 130 contour points as shown in Fig. 1b. Since AP pelvic radiographs only give a two-dimensional projection of the three-dimensional proximal femur, building a combined model allowed us to analyse whether the projections of the left and right proximal femurs varied in a symmetrical manner. The aim was to identify whether there were any oppositional patterns of asymmetric shape variation i.e. patterns where the shape variation between the left and right sides were similar but in opposite directions. Modes of variation that were clearly attributable to an oppositional asymmetric pattern of shape variation between the left and the right sides (e.g. due to subject positioning during image acquisition) were excluded. This was achieved by setting the relevant shape model modes in ***b*** to zero (see Eq. (1)) and subsequent re-evaluation of all 130 points ***x***_*l*_ for every subject. To analyse the difference in shape between the point positions identified by the *FASMM* system and the point positions after exclusion of specific modes, we aligned the two point sets for every subject using a similarity transformation and calculated the mean point-to-curve distance over all 130 points. We used custom code developed in C++ for this analysis and to generate the graphics.

### Analysing global proximal femur symmetry using SSMs

For both the original point positions as well as the re-evaluated point positions (i.e. after adjusting for subject positioning), we built a single proximal femur SSM using 65 points each as shown in Fig. 1a. We included the left and the right proximal femurs of every subject separately into each SSM. For the purpose of this analysis, we made all femurs appear as left femurs (i.e. right femurs were reflected accordingly). We then used the single proximal femur SSMs to analyse proximal femur shape variation between contra-lateral hips, before and after adjusting for oppositional asymmetric patterns of shape variation. To determine the average shape difference between the left and right sides, we calculated the mean point-to-curve distance over all 65 points between the left and right femurs of every subject. We also compared the standard deviation of the shape variation between the left and right femurs to the overall shape variation in the population for every shape model mode of each SSM. A univariate independent two-sample Welch's t-test on the SSM mode values was used to investigate whether there was a significant difference in radiographic proximal femur shape between the left and the right femurs of all subjects. The mean shape variation was calculated across all images and then individually for the left and right subgroups. All plots and calculations were made using custom code developed in C++ and Matlab R2010a.

### Analysing the impact of subject-positioning on hip geometric measurements

To analyse the symmetry of the left and right proximal femurs when using conventional hip geometric measurements *before* and *after* adjusting for oppositional asymmetric patterns of shape variation, we fully automatically calculated hip geometric measurements from the output of the *FASMM* as previously described [20]. We obtained the femoral head diameter, neck width, shaft width and neck-shaft angle for both hips of every subject. The formula for calculating the absolute percent asymmetry (AA%) for each measurement and every subject was:

(2) $$\mathrm{AA\%}=\frac{\left|m_{l}-m_{r}\right|}{\mu }x100\;$$

where *m*_*l*_ and *m*_*r*_ are the measurement values for the left and right sides of the subject and *μ* is the mean of the left and right measurement values for that subject. All calculations were made using custom code developed in Matlab R2010a (see [20]).

## Results

### Performance of the *FASMM* system

The fully automatic segmentation of the 1282 radiographs using the *FASMM* system took on average 1 min per radiograph per hip joint. The *FASMM* system accurately segmented the left and right proximal femurs in 1258 of these radiographs. Of the remaining 24 images, in 21, one of the femurs was not outlined accurately and in 3, both femurs were not outlined correctly, mainly due to very poor image contrast. This gave a segmentation accuracy of 99% based on single femurs and 98% based on full pelvic images.

The analyses described below were conducted using the accurately segmented subset of 1258 radiographs (mean age: 61.2 SD = 9.0; mean BMI: 27.3 SD = 4.9).

### Adjusting for subject positioning

**Fig. 2:**
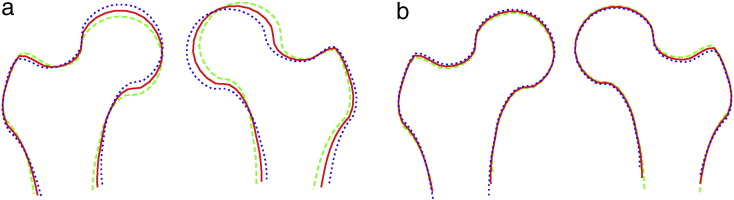
Two modes of the combined proximal femur model show clear oppositional asymmetric patterns of shape variation for the left and right proximal femurs: (a) Mode 3 — global shape differences; (b) Mode 6 — local shape differences along the femoral shaft. Each figure shows the average () and ± 2.5 standard deviations.

**Fig. 3:**
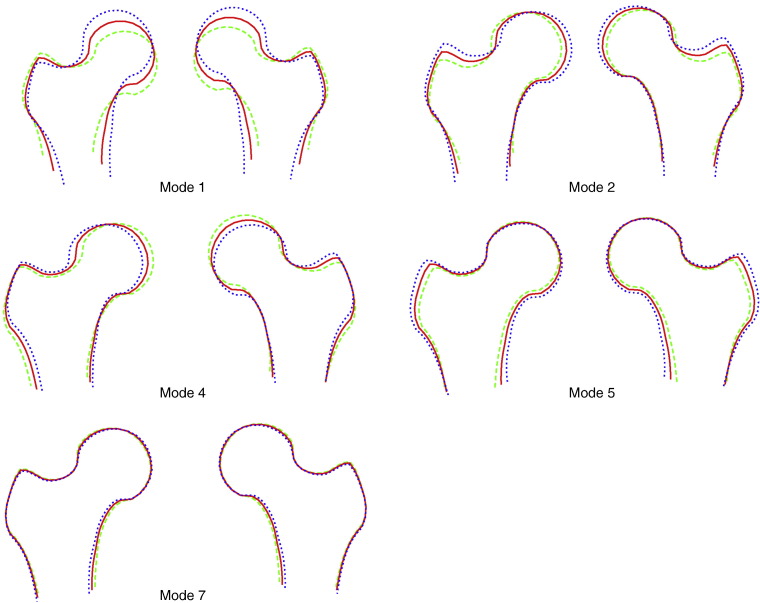
Modes 1, 2, 4, 5 and 7 of the combined proximal femur model show approximately symmetric patterns of shape variation for the left and right proximal femurs (modes 1–7 explained 85% of overall shape variation in the combined proximal femur model). Each figure shows the average () and ± 2.5 standard deviations.

For the combined SSM, 95% of the overall shape variation was given by 16 modes. Two of these modes showed clear oppositional asymmetric patterns of shape variation for the left and right proximal femurs as visualised in Fig. 2. For comparison, five additional modes of the combined SSM are illustrated in Fig. 3. Together these first seven modes (i.e. the two illustrated in Fig. 2 and the five illustrated in Fig. 3) of the combined SSM explained 85% of overall shape variation.

As shown in Fig. 2a, mode 3 demonstrated *global* shape differences between the left and the right proximal femur. The orientation of the femoral shaft and femoral head on the left and the right changed in an oppositional asymmetric manner. For example, when the right femoral head was more superior and the right femoral shaft more medially orientated the opposite held true for the left femur. As shown in Fig. 2b, mode 6 demonstrated *local* shape differences between the left and the right proximal femurs in the area of the femoral shaft. The position of the points that outlined the femoral shaft slid up and down the shaft in an oppositional asymmetric manner. For example, when the points outlining the femoral shaft of the right femur were stretched down the shaft, the same points of the left femur were not and *vice versa*.

**Fig. 4:**
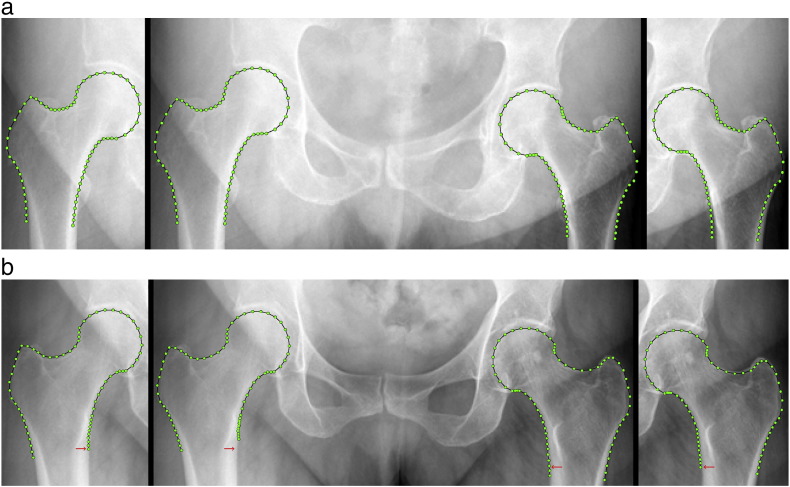
The effect of excluding modes 3 and 6. The point positions identified by the *FASMM* system (prior to exclusion of modes 3 or 6) are given in the centre of each image and the updated point positions after adjustment are given to the left and right: (a) exclusion of mode 3 (difference in mean point-to-curve distance between original and re-evaluated point positions for the subject shown: 1.8 mm); (b) exclusion of mode 6 (difference in mean point-to-curve distance between original and re-evaluated point positions for the subject shown: 0.8 mm). To aid comparison, the red arrow indicates the same position before and after exclusion of mode 6.

Fig. 4 shows the effect of excluding mode 3 or mode 6 from the SSM. As shown in Fig. 4a, the updated point positions after excluding mode 3 do not alter the overall shape but adjust the orientation of the femurs to account for the orientation of the pelvis. As shown in Fig. 4b, the updated point positions after excluding mode 6 demonstrate a ‘shaft sliding’ effect i.e. the points along the femoral shaft differ in position along the shaft of the bone relative to the position of the lesser trochanter. Of note is that ‘shaft sliding’ does not necessarily occur on both the left and right sides as it does in the example in Fig. 4b but can also occur on one side only.

We then excluded modes 3 and 6 and re-evaluated all point positions for all subjects. By excluding mode 3 only, the *average* shape difference between the point positions identified by the *FASMM* system and the point positions after re-evaluation was 0.6 mm (mean point-to-curve distance over all 130 points and all subjects). In comparison, when excluding mode 6 only the *average* shape difference between the point positions identified by the *FASMM* system and the point positions after re-evaluation was 0.2 mm (mean point-to-curve distance over all 130 points and all subjects). This arose as ‘shaft sliding’ occurred only for a minority of the 1258 radiographs used whereas the *global* shape differences as represented by mode 3 of the combined SSM were more prevalent. For 95% of the images the shape difference between the automatically identified point positions and the point positions after re-evaluation was within 1.5 mm after excluding mode 3 only and within 0.5 mm after excluding mode 6 only. When excluding both modes 3 and 6, the *average* shape difference between the point positions identified by the *FASMM* system and the point positions after re-evaluation was 0.7 mm (mean point-to-curve distance over all 130 points and all subjects). This analysis thus identified that the majority of oppositional asymmetric shape variation between the left and right proximal femurs was attributable to mode 3.

### Analysing global proximal femur symmetry using SSMs

**Fig. 5:**
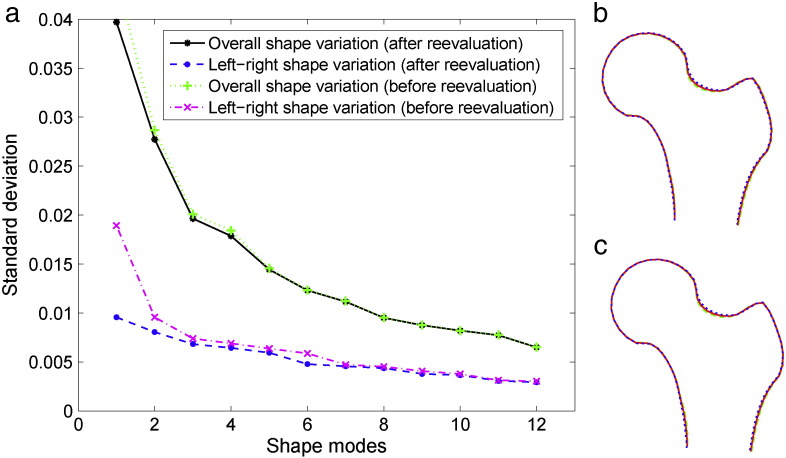
Analysis of proximal femur symmetry (using the left and right femurs of 1258 Caucasian females from the OAI dataset): (a) standard deviation of the overall shape model modes compared to the standard deviation of the within person left–right shape variation before and after adjusting for oppositional asymmetric patterns of shape variation; (b)–(c) shape differences between right () and left () proximal femur mean shapes before and after adjusting for oppositional asymmetric patterns of shape variation where differences from the overall mean () have been exaggerated by a factor of 10 to aid visualisation.

The analysis of global femur morphology based on the single proximal femur SSMs of all 1258 subjects showed that the average shape difference between the left and the right proximal femurs was 1.0 mm before and 0.8 mm after the exclusion of modes 3 and 6 (mean point-to-curve distance over all 65 points and all subjects). Fig. 5a shows that this difference was small in comparison to the overall shape variation in the population. This was supported by the difference between left and right proximal femur mean shapes as shown in Fig. 5b before and Fig. 5c after re-evaluation of point positions where the differences from the overall mean have been exaggerated by a factor of 10 to aid visualisation.

Both of the *single* proximal femur SSMs explained 95% of the overall shape variation given by the dataset using 12 modes. SSM mode values for each of the 1258 subjects were calculated and the mean SSM values for each of the 12 modes and each of the groups (i.e. left vs. right proximal femurs) were compared using a univariate independent two-sample Welch's t-test. We used the Kolmogorov–Smirnow test to verify that the data for each mode and every subject group were normally distributed. Before and after adjusting for oppositional asymmetric patterns of shape variation, only two of the 12 modes (modes 6 and 7) had left vs. right mean values that were significantly different (p < 0.05). After Bonferroni adjustment, only one of the 12 modes (mode 6) had left vs. right mean values that were significantly different (p < 0.004) before and after adjustment.

### Analysing the impact of subject-positioning on hip geometric measurements

When using the fully automatically obtained point positions (after adjustment for oppositional asymmetry) to automatically calculate conventional hip geometric measurements, the average absolute difference for all measurements between the left and right hip joints of each individual was within 1.1 mm or 2.9° and no measurement exceeded 1.2 mm or 3.1° at the 95% confidence interval (see Table 2). The average absolute percent asymmetry was within 3.1% for all measurements with all 95% confidence intervals falling between 1.5% and 3.2%. We obtained similar results when using the automatically obtained point positions *before* adjustment for oppositional asymmetry to automatically calculate conventional hip geometric measurements. As is evident in Table 2, the overall values did not significantly differ *before* and *after* adjustment for oppositional asymmetric patterns of shape variation. In terms of symmetry between the left and right hip joints, the femoral head diameter and neck width did not change following the adjustment of point positions. A significant decrease in absolute difference and absolute percent asymmetry before vs. after adjustment was, however, identified for the shaft width and the neck-shaft angle.

## Discussion

We have investigated the symmetry of the left and right proximal femurs in AP pelvic radiographs of female subjects. We have shown that the *global* shape of the left and right proximal femurs is symmetric without isolated locations of asymmetry. We have demonstrated that a combined SSM of the left and right proximal femurs can be used to identify and adjust for oppositional asymmetric patterns of shape variation. We have also shown that radiographic proximal femur symmetry increases after adjusting for these patterns of oppositional asymmetric shape variation when analysed using both SSM-based and conventional hip geometric measurements.

When we analysed the combined SSM, we identified two shape modes, modes 3 and 6, that showed oppositional patterns of asymmetric shape variation between the left and right femurs (see Fig. 2). Mode 3 is characterised by an oppositional change in the orientation of the proximal femur. Analysing mode 3 in more detail indicated that this mode is linked to pelvic rotation as illustrated in Fig. 4a. As shown in this figure, the overall shape does not change but the orientation of the femurs has been altered according to the orientation of the pelvis. We hence believe that mode 3 is attributable to subject positioning during image acquisition. Mode 6 demonstrates ‘shaft sliding’. As discussed in [17], this sliding of the points along the femoral shaft is related to the visibility of the lesser trochanter since the *FASMM* system uses information about the appearance of the lesser trochanter for the positioning of the points along the shaft. Shaft sliding occurs for radiographs where the lesser trochanter is obscured by the femoral shaft. This again is attributable to subject positioning during image acquisition as the appearance of the lesser trochanter in an AP pelvic radiograph depends on the internal/external rotation of the leg. Fig. 4b gives an example where exclusion of mode 6 counteracts shaft sliding.

The proposed approach to adjust for subject positioning mainly addresses rotation within the transverse and coronal planes, and not the sagittal plane. However, it appears that rotation within the sagittal plane during image acquisition of an AP pelvic radiograph has the smallest effect on changes in the two-dimensional projection of proximal femur shape when compared to rotation within the transverse and coronal planes.

When comparing conventional hip geometric measurements before and after the re-evaluation of point positions (i.e. exclusion of modes 3 and 6), we found that there was a significant decrease in the absolute difference between the measurements for the left and right femurs as well as in the absolute percent asymmetry for both the shaft width and the neck-shaft angle (see Table 2). We believe that the difference in shaft width is mainly due to adjusting for shaft sliding (which was achieved by excluding mode 6 from the combined SSM). Similarly, we believe that the difference in neck-shaft angle is mainly due to adjusting for pelvic rotation (which was achieved by excluding mode 3 from the combined SSM). The findings obtained when comparing hip geometric measurements support the assumption that reevaluating point positions after excluding modes of oppositional asymmetric patterns in the combined SSM is a suitable approach to account for subject positioning during image acquisition. Hence, we consider that the measurements obtained after adjusting for oppositional asymmetric patterns of shape variation are more representative of actual shape. However, excluding modes from the combined SSM needs to be approached with caution. It is important to only exclude modes that clearly show *oppositional* asymmetric patterns of shape variation between the left and right proximal femurs to avoid removal of true shape differences between the left and right proximal femurs. Hence, when building a combined SSM to adjust for subject positioning, it is necessary to use a large dataset of at least several hundred images such that an accurate and comprehensive SSM can be generated. It is also necessary to minimise shape heterogeneity within the dataset as this allows a clearer distinction between shape modes that represent actual anatomic shape variation and shape modes that represent oppositional patterns of asymmetric shape variation due to subject positioning. Since it is known that there are anatomical differences in hip joint shape between genders and across ethnic groups, and as the combined SSM indirectly encodes pelvic shape via the relative position of the left and right proximal femurs, we reduced shape heterogeneity in the generation of the combined SSM by using a single-gender single-ethnicity dataset. Hence, we restricted the dataset to Caucasian females which represented the largest proportion of the dataset. We built the single SSM that was used for the analysis of proximal femur shape variation between contra-lateral sides using the same Caucasian female subgroup to allow the analysis of proximal femur symmetry *before* and *after* adjusting for oppositional asymmetric patterns of shape variation.

We used two different approaches to analyse proximal femur morphology: statistical shape models and conventional hip geometric measurements. The first approach used the more complete information of hip shape and allowed the analysis of *global* shape variation across a population. For the second approach, we selected measurements from the literature that have been previously used to analyse radiographic proximal femur symmetry. Proximal femur symmetry has not been analysed before using SSMs. Therefore, we show for the first time that the *global* shape of the left and right proximal femurs is symmetric in a Caucasian female population. When using conventional hip geometric measurements in our large dataset, our findings were similar to those published previously [4,5] suggesting that the geometry of the left and right proximal femurs is not significantly different. As in [4], we also found that the absolute percent asymmetry did not exceed 4% for any of the measurements. However, when using both of the above approaches we found that left–right femur symmetry was increased after adjusting for subject positioning during image acquisition based on the identification of oppositional asymmetric patterns of shape variation.

The main limitation of this study is that it relies on two-dimensional AP pelvic radiographs which only give a two-dimensional projection of the three-dimensional proximal femur. Hence, *(i)* the projected radiographic shape of the proximal femur may vary due to subject positioning during image acquisition and *(ii)* a single AP view is not a full representation of the three-dimensional structure. To counteract the former, we investigated how oppositional asymmetric patterns of shape variation in a combined left–right SSM can be used to account for pelvic and internal/external leg rotation during image acquisition. In terms of the limited information in an AP pelvic radiograph, further studies may be required on additional two-dimensional views (e.g. frog-leg lateral) or even CT data to confirm three-dimensional symmetry of the left and right proximal femurs. However, AP pelvic radiographs are widely used in clinical practise and thus this study provides broad applicability. In addition, recent research has shown that some three-dimensional measurements as obtained from CT data can be predicted from AP pelvic radiographs [28].

This analysis was done using a front-view femur model that excludes both the lesser and greater trochanters. The captured shape is sufficient to derive hip geometric measurements such as the head diameter, neck width, or neck-shaft angle. However, in future, we aim to extend the *FASMM* system to also include the trochanters and the pelvis. Furthermore, this study was performed using Caucasian female subjects only. Although it has been previously shown that the shape of male and female proximal femurs differ, using either SSMs or conventional geometric measurements [6,20], there is no evidence that male left–right symmetry differs from that of females. There is also no evidence that left–right symmetry differs according to ethnicity. Absolute confirmation of this would, however, require the analyses described in this paper to be repeated on these datasets. In our study, we have also not addressed the potential causes of positional shape variation which may well be related to disease (e.g. due to knee pain in subjects with knee osteoarthritis). Further work would be required in order to identify whether there was an association between subject positioning and disease status or progression.

## Conclusions

Our findings demonstrate that the *global* shape of the left and right proximal femurs as represented by AP pelvic radiographs of Caucasian female subjects is symmetric without isolated locations of asymmetry and that using a combined left–right SSM (on a sufficiently large dataset) for adjustment of subject positioning during image acquisition improves the accuracy of predicting the shape of the contra-lateral hip joint. When deriving conventional hip geometric measurements using the point positions returned by the *FASMM* system our symmetry results were similar to previously published results. This study also demonstrates that our *FASMM* system (which is freely available for non-commercial research purposes) is a time-efficient and effective way to analyse global shape variation across large datasets, having implications not only for orthopaedic surgery planning but also for large scale analyses of bone shape variation and disease associations.

## Figures and Tables

**Table 1:** Summary statistics for the 1282 subjects included in this study (mean age: 61.3 SD = 9.0; mean BMI: 27.3 SD = 5.0).

	# Subjects out of 1282	
	At baseline	At 48 months
Any hip pain, aching or stiffness in past 12 months	741	660
Infrequent knee pain	492	566
Frequent knee pain	534	486
Self-reported knee osteoarthritis	234	288
Self-reported rheumatoid arthritis	50	54

**Table 2:** Analysis of proximal femur symmetry based on 1258 Caucasian females from the OAI dataset (mean age: 61.3 SD = 9.0; mean BMI: 27.3 SD = 4.9): measurement values, absolute differences and absolute percent asymmetry for left vs. right proximal femurs.

Measurement	Values a	95% CI	AD a,b	95% CI	AA% a,c	95% CI
***Before adjusting for oppositional asymmetric patterns of shape variation***						
Head diameter (mm)	51.6 ± 3.4	(51.4,51.8)	0.8 ± 0.7	(0.7,0.8)	1.5 ± 1.3	(1.4,1.6)
Neck width (mm)	35.3 ± 2.9	(35.2,35.5)	0.9 ± 0.8	(0.8,0.9)	2.5 ± 2.1	(2.4,2.6)
Shaft width (mm)	37.0 ± 3.2	(36.9,37.2)	1.6 ± 1.3	(1.5,1.6)	4.2 ± 3.5	(4.0,4.4)
Neck-shaft angle (°)	126.7 ± 5.8	(126.4,127.0)	3.6 ± 2.8	(3.4,3.7)	2.8 ± 2.2	(2.7,2.9)

***After adjusting for oppositional asymmetric patterns of shape variation***						
Head diameter (mm)	51.5 ± 3.4	(51.3,51.7)	0.8 ± 0.7	(0.8,0.8)	1.5 ± 1.3	(1.5,1.6)
Neck width (mm)	35.3 ± 2.9	(35.1,35.5)	0.9 ± 0.8	(0.9,1.0)	2.6 ± 2.1	(2.5,2.7)
Shaft width (mm)	37.0 ± 3.1	(36.8,37.2)	1.1 ± 0.9	(1.1,1.2)	3.1 ± 2.3	(2.9,3.2)
Neck-shaft angle (°)	126.8 ± 5.7	(126.5,127.1)	2.9 ± 2.4	(2.8,3.1)	2.3 ± 1.9	(2.2,2.4)

a The data are given as mean ± SD.

b AD = absolute difference between left and right.

c AA% = absolute percent asymmetry.
